# The role of ecotypic variation in driving worldwide colonization by a cosmopolitan plant

**DOI:** 10.1093/aobpla/ply005

**Published:** 2018-01-24

**Authors:** Barbara Neuffer, Christina Wesse, Ingo Voss, Renate Scheibe

**Affiliations:** 1 Department of Botany, University of Osnabrück, Barbarastr., Osnabrück, Germany; 2 Department of Plant Physiology, University of Osnabrück, Barbarastr., Osnabrück, Germany

**Keywords:** Anatomy, *Capsella*, chlorophyll fluorescence parameters, leaf types, photosynthetic capacity, Shepherd’s Purse

## Abstract

For almost 100 years now, ecotypic differentiation of plant species has been a major topic of research. In changing environments, the question needs to be answered as to how long it takes to adapt, and which parameters are subject to this fast adaptation. Short-living colonizing plant species are excellent examples, especially when they are selfing. Shepherd’s Purse *Capsella bursa-pastoris* (Brassicaceae) is one of the most wide-spread flowering species on earth and avoids only the hot and humid tropics. Many studies demonstrated the ecotypic differentiation of *C. bursa-pastoris* in various regions of the world but ecotypic differentiation regarding adaptability of anatomy and physiology of rosette leaves so far remained less recognized. However, the leaves are relevant for subsequent seed set; in particular, winter-annual accessions require a robust rosette to survive adverse conditions. Leaf-related traits such as the thickness of the mesophyll and epidermis, stomatal density, photosynthetic capacity and the ability to withstand and even use high light conditions were therefore analysed in provenances from various climatic zones. Photosynthetic capacity depends on leaf anatomy and cellular physiological parameters. In particular, the ability to dynamically adjust the photosynthetic capacity to changing environmental conditions results in higher fitness. Here, we attempt to relate these results to the four Mendelian leaf types according to Shull.

## Teaser

Short-living plants meet their main energy demand by photosynthesis of rosette leaves. These leaves optimize their yield as required for successful growth in so many different locations around the world. Do they cope with varying conditions by one general-purpose genotype and phenotypic plasticity or by ecotypic differentiation with a specific genetic background? How did leaves of a plant like Shepherd’s Purse, a weed colonizing all continents and climatic regions during a time period of only 500 years, evolve? We demonstrate that a combination of physiological, anatomical and morphological differentiation might allow such tremendous colonization success.

## Introduction

Since Turesson’s pioneering work (1922a, b,^1930^), the ecotypic differentiation of plant species has been a main interest in population biology: how and how quickly are plant species able to adapt to changing environmental conditions. Due to their property of self-fertilization, short-living colonizing plant species are suitable examples. A single seed is able to colonize a new habitat and to establish a new population. Which traits play a role in this highly successful colonizing history? For a short-living plant, several characters are critical, in particular, the physiology of germination and the determination of flowering time. In both developmental steps, the plant quits a status that is resistant to harsh conditions and changes to a highly sensitive status. If the environment is unfavourable, the individual will not complete its life cycle or it will produce only a low amount of immature seeds. Between germination and flowering, when they grow vegetatively in a rosette status, these plants are able to survive extreme conditions, e.g. cold winter. So far, numerous investigations have aimed at the control of flowering time, but less information is available concerning ecotypic differentiation with regards to a combination of morphological, anatomical and physiological leaf characters, which we are focussing on in this study.

The Shepherd’s Purse *Capsella bursa-pastoris* (Brassicaceae) belongs to the most prevalent flowering plants on earth (Coquillat 1951; Zhou *et al.* 2001), but it is not found in the hot and humid tropics. Their extraordinary colonizing success may be caused by the predominantly selfing mating system, rapid propagation by seeds as an annual to winter annual, the production of an enormous amount of seeds per individual (Hurka and Neuffer 1991), the ability to survive in a soil seed bank for many years (Hurka and Haase 1982) and the power for long-distance dispersal via myxospermy (Neuffer and Linde 1999).

In prehistoric times *C. bursa-pastoris* was distributed over the whole of Eurasia including the regions surrounding the Mediterranean Sea either along river shores or by early agricultural activities of humans. Later on, from the beginning of the 16th century, Europeans colonized all other continents, used the same agricultural techniques and crop plants and introduced many weeds as neophytes that in some cases turned out to be pests for the native biodiversity (e.g. Mooney *et al.* 2005). This unintentional transport paved the way for *C. bursa-pastoris* to reach the New World, Australia, South Africa, New Zealand, the Falkland Islands and other localities (Neuffer and Hurka 1999; Neuffer *et al.* 1999,^2011^; Kryvokhyzha *et al.* 2016). This fast expansion of one weedy plant species was only possible due to its extraordinary capability of ecotypic differentiation. The differentiation of *C. bursa-pastoris* has been recorded for Europe (e.g. Neuffer and Bartelheim 1989, reviewed in Neuffer 2011), and such preadapted ecotypes have been able to find their niche elsewhere on the globe (Neuffer and Hurka 1999; Neuffer *et al.* 1999).

In many studies, the ecotypic differentiation of *C. bursa-pastoris* has been demonstrated for various regions of the world predominantly regarding germination, flowering time, rosette diameter and number of inflorescence branches (reviewed in Neuffer *et al.* 2011). Adaptive traits are frequently related to the rosette leaves that are responsible for the production of resources required for subsequent yield and abundant seed set. In the case of winter annuals, a robust rosette is required to survive suboptimal weather conditions. Important leaf traits are rosette diameter, the number of leaves in a rosette, leaf area, thickness of the leaf as well as of the epidermal cells, stomatal density, and photosynthetic capacity, as well as photosynthetic light utilization. Stomatal density and other epidermal characteristics strongly influence water-use efficiency (WUE), which is particularly important in dry habitats accompanied by high irradiation (reviewed in Körner 2003). The photosynthetic capacity depends on number, total area and anatomy of leaves and on cellular physiological parameters; in particular, the ability to dynamically adjust photosynthetic capacity to changing environmental conditions results in higher fitness (Athanasiou *et al.* 2010). The climatic adaptability and ecotypic intraspecies differentiation in a combination of morphological, anatomical and physiological characters have been shown for *Diplotaxis erucoides* populations from Sicily by Schleser *et al.* (1989).

**Figure 1.**
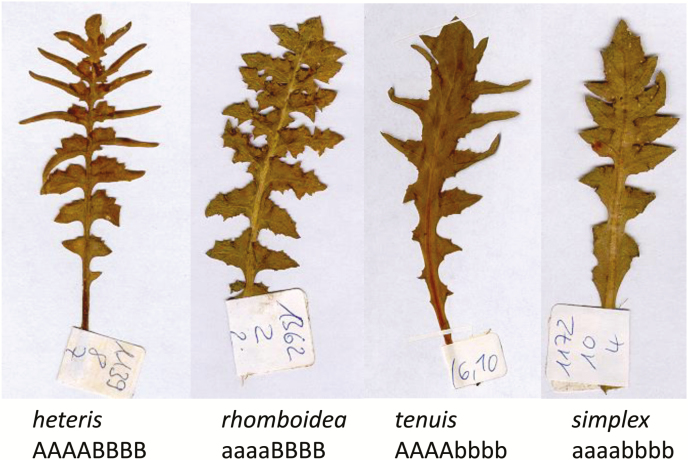
Rosette leaf types and allele formulas of tetraploid *Capsella bursa-pastoris* after Shull.

The degree of leaf-margin dissection—from entire leaves with smooth margins to serrated and increasingly deeply lobed leaves—is likely to be functionally important. Leaf-margin dissection shows a very robust negative correlation with mean annual temperature, both at the within-species and at the community level. Such a wide-spread relationship between a morphological trait and an environmental parameter across different phylogenetic scales provides a strong argument that the trait in question is adaptive (Nicotra *et al.* 2011). Recently, the analysis of leaf size and compound leaves of a large number of species in relation to geography and climate was analysed by Wright *et al.* (2017) which is an indicator for global climatic change. The genus *Capsella* shows a high level of variation in all of the above-mentioned leaf-related traits, including the leaf shape, which ranges from entire leaves to very deeply dissected ones (Fig. 1; Shull 1909; Sicard *et al.* 2014).

Here, we will shed light on a combination of morphological/anatomical and physiological variation that underlies rapid local adaptation in one of the world’s most successful weeds.

## Methods

For each of the four data sets different treatments and determinations were performed as described below. It was our aim to analyse all leaves at the same stage of development. Therefore, we started the leaf studies when the first flower bud appeared indicating the end of the vegetative phase. In the case of late flowering plants, we started no later than 3 months after sowing to avoid any senescence.

### Analysis 1: isotope analysis

**Figure 2.**
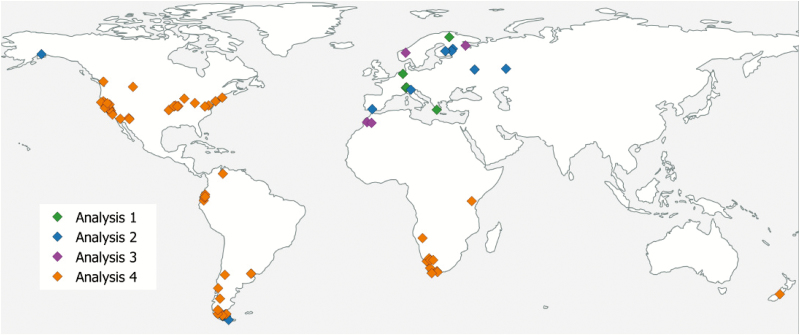
Localities of provenances used in four different experimental designs (Analyses 1–4). Analysis 1: six populations (green colour); Analysis 2: 15 populations (blue colour), Analysis 3: four populations (purple colour); Analysis 4: 76 populations (orange colour).

To test the ability of progenies from different environmental habitats to react on drought stress we grew sister individuals under different conditions and analysed δ^13^C values in combination with morphological and anatomical features (Fig. 2, green colour; Table 1). Plants were grown in a growth chamber with a day-night rhythm of 15:9 h with 15–25 °C. Progeny of individuals collected in the wild (Fig. 2) were divided into two groups, one group was kept under water stress conditions with a maximum of 10 mL water daily per 1-L pot, the other group with at least 30 mL daily, for non-stress conditions. Each individual was planted in 1-L substrate with sand and slightly fertilized turf in a proportion of 1:2. Each population and each condition were represented by up to five sister individuals. For each individual (three individuals for each population and treatment), cell size, stomata density and the percentage of the volume of mesophyll cells compared with intercellular space were measured 30 times. Furthermore, the δ^13^C values were determined as follows: dried leaf material was combusted in an excess of oxygen at ~1000 °C, and the resulting CO_2_ used for isotope analysis using the MAT 250 mass spectrometer (Schleser and Poling 1980). Carbon isotope values of leaves are based on total organic matter rather than on a selected chemical compound such as cellulose. Several test measurements have shown that relative variations of the isotope content led to similar results for total organic matter and cellulose. Only the absolute values differ by 2 to 2.5 °/_00_. Results are reported in terms of δ^13^C relative to PDB (Belemnite from the Pee Dee formation in South Carolina; Craig 1957). The δ^13^C value as the ratio between ^13^CO_2_ and ^12^CO_2_ was measured twice for each individual.

**Table 1.** Provenances of all studied *Capsella* populations; country labelled by KFZ.

Population	Country	Locality	Latitude	Longitude	Elevation (m)	Species	Leaf type after Shull	Collector	Analysis
83	D	Teglingen	52,65	7,35	14	*C. bursa-pastoris*	*rhomboidea*	Benneweg	1
147	FIN	Ivalo	68,65	27,57	160	*C. bursa-pastoris*	*rhomboidea*	Bosbach, K., Hurka, H.	1
257	CH	Disentis	46,68	8,83	1400	*C. bursa-pastoris*	*rhomboidea*	Hurka, H.	1
279	CH	Andermatt	46,63	8,6	1480	*C. bursa-pastoris*	*tenuis*	Hurka, H.	1
282	CH	Trun	46,75	8,98	850	*C. bursa-pastoris*	*heteris*	Hurka, H.	1
434	GR	Kalamata	37,04	22,12	1270	*C. rubella*	*simplex*	Bosbach, K., Hurka, H.	1
679	USA	Neosho	36,87	−94,37	323	*C. bursa-pastoris*	*sim/rho*	Borgwart, M.	4
680	USA	Neosho	36,87	−94,37	323	*C. bursa-pastoris*	*tenuis*	Borgwart, M.	4
681	USA	Chicago	41,88	−87,63	182	*C. bursa-pastoris*	*heteris*	Borgwart, M.	4
700	USA	Davis	38,53	−121,73	16	*C. bursa-pastoris*	*heteris*	Hurka, H.	4
701	USA	Davis	38,53	−121,73	16	*C. bursa-pastoris*	*sim/rho*	Hurka, H.	4
702	USA	Davis	38,53	−121,73	16	*C. bursa-pastoris*	*tenuis*	Hurka, H.	4
703	USA	Davis	38,53	−121,73	16	*C. bursa-pastoris*	*rhomboidea*	Hurka, H.	4
706	USA	Davis	38,53	−121,73	16	*C. bursa-pastoris*	*simplex*	Hurka, H.	4
712	USA	Williams	39,15	−122,15	25	*C. bursa-pastoris*	*sim/rho*	Hurka, H.	4
713	USA	Stockton	37,95	−121,28	5	*C. bursa-pastoris*	*simplex*	Hurka, H.	4
714	USA	Stockton	37,95	−121,28	5	*C. bursa-pastoris*	*tenuis*	Hurka, H.	4
715	USA	Coulterville	37,72	−120,2	544	*C. bursa-pastoris*	*rhomboidea*	Hurka, H.	4
717	USA	Fresno	36,75	−119,77	98	*C. bursa-pastoris*	*tenuis*	Hurka, H.	4
718	USA	Fresno	36,57	−119,62	98	*C. bursa-pastoris*	*sim/ten*	Hurka, H.	4
722	USA	Shafter	35,5	−119,27	106	*C. bursa-pastoris*	*simplex*	Hurka, H.	4
723	USA	Wheeler Ridge	34,98	−118,93	111	*C. bursa-pastoris*	*simplex*	Hurka, H.	4
726	USA	Tuttle	37,3	−120,38	62	*C. bursa-pastoris*	*simplex*	Hurka, H.	4
727	USA	Willows	39,52	−122,3	67	*C. bursa-pastoris*	*heteris*	Hurka, H.	4
728	USA	Willows	39,52	−122,2	43	*C. bursa-pastoris*	*simplex*	Hurka, H.	4
729	USA	Chico	39,78	−121,95	59	*C. bursa-pastoris*	*simplex*	Hurka, H.	4
730	USA	Red Bluff	40,15	−122,25	103	*C. bursa-pastoris*	*heteris*	Hurka, H.	4
732	USA	Douglas City	40,65	−122,93	609	*C. bursa-pastoris*	*ten/rho*	Hurka, H.	4
733	USA	Weaverville	40,73	−122,93	636	*C. bursa-pastoris*	*ten/het*	Hurka, H.	4
736	USA	Myers Flat	40,27	−123,87	85	*C. bursa-pastoris*	*sim/het*	Hurka, H.	4
745	USA	Placerville	38,73	−120,67	610	*C. bursa-pastoris*	*rhomboidea*	Hurka, H.	4
746	USA	Davis	38,53	−121,73	16	*C. bursa-pastoris*	*sim/rho*	Hurka, H.	4
747	USA	Truckee	39,33	−120,18	1819	*C. bursa-pastoris*	*sim/ten*	Hurka, H.	4
748	USA	Berkeley	37,87	−122,25	112	*C. bursa-pastoris*	*simplex*	Hurka, H.	4
750	USA	Bucks Lake	39,87	−121,17	1582	*C. bursa-pastoris*	*sim/het*	Hurka, H.	4
785	USA	Jefferson City	38,52	−92,07	207	*C. bursa-pastoris*	*sim/ten*	Koch	4
786	USA	Montgomery City	38,88	−91,45	266	*C. bursa-pastoris*	*ten/het*	Koch	4
846	USA	St Louis	38,63	−90,18	141	*C. bursa-pastoris*	*tenuis*	Neuffer, B.	4
847	USA	Jefferson City	38,57	−92,17	194	*C. bursa-pastoris*	*simplex*	Neuffer, B.	4
848	USA	St Louis	38,5	−90,63	191	*C. bursa-pastoris*	*tenuis*	Neuffer, B.	4
852	USA	Boston	42,35	−71,07	5	*C. bursa-pastoris*	*rho/het*	Neuffer, B.	4
853	USA	Boston	42,35	−71,07	5	*C. bursa-pastoris*	*sim/het*	Neuffer, B.	4
855	USA	Columbus	39,95	−83	230	*C. bursa-pastoris*	*simplex*	Crawford, D.J.	4
939	YV	Pico el Aguila	8,85	−70,82	3877	*C. bursa-pastoris*	*simplex*	Bosbach, K.	4
961	NAM	Etosha National Park	−19,17	15,92	1178	*C. bursa-pastoris*	*simplex*	Schröpfer, R.	4
966	EAT	Mt. Kilimanjaro Nat. Park	−3,07	37,37	5325	*C. bursa-pastoris*	*simplex*	Hurka, H.	4
1137	FIN	Nurmes	63,55	29,12	120	*C. bursa-pastoris*	*rhomboidea*	Neuffer, B.	2
1139	FIN	Kuopio	62,58	28,59	95	*C. bursa-pastoris*	*heteris*	Neuffer, B.	2
1141	FIN	Suolahti	62,57	25,85	100	*C. bursa-pastoris*	*heteris*	Neuffer, B.	2
1141	FIN	Suolahti	62,57	25,85	100	*C. bursa-pastoris*	*tenuis*	Neuffer, B.	2
1198	RCH	Puerto Octay	−41	−72,88	153	*C. bursa-pastoris*	*sim/ten*	Hurka, H.	4
1273	E	Pilas	37,3	−5,7	80	*C. bursa-pastoris*	*simplex*	Neuffer, B.	2
1355	I	Malcesine	45,77	10,82	800	*C. bursa-pastoris*	*simplex*	Neuffer, B.	2
1357	USA	Anchorage	61,22	−149,88	20	*C. bursa-pastoris*	*simplex*	Handke	2
1376	RA	Buenos Aires	−34,67	−58,5	19	*C. bursa-pastoris*	*tenuis*	Damborenea, S.	4
1377	RA	Buenos Aires	−34,67	−58,5	10	*C. rubella*	*heteris*	Damborenea, S.	2
1377	RA	Buenos Aires	−34,67	−58,5	19	*C. bursa-pastoris*	*rhomboidea*	Damborenea, S.	4
1380	RCH	Punta Delgada	−52,45	−69,55	50	*C. bursa-pastoris*	*heteris*	Neuffer & Neuffer	4
1381	RCH	San Sebastian	−53,15	−69,4	100	*C. bursa-pastoris*	*heteris*	Neuffer & Neuffer	2
1385	RA	Ushuaia	−54,8	−68,3	20	*C. bursa-pastoris*	*tenuis*	Neuffer & Neuffer	2
1387	RCH	Punta Delgada	−52,22	−69,28	200	*C. bursa-pastoris*	*ten/rho*	Neuffer & Neuffer	4
1388	RCH	Porto Gregorio	−52,32	−69,74	10	*C. bursa-pastoris*	*heteris*	Neuffer & Neuffer	2
1389	RCH	Punta Arenas	−52,9	−70,97	34	*C. bursa-pastoris*	*heteris*	Neuffer & Neuffer	4
1390	RCH	Tehuelche	−53,15	−70,89	200	*C. bursa-pastoris*	*tenuis*	Neuffer & Neuffer	2
1393	RCH	Nationalpark Torres del Paine	−50,72	−72,7	578	*C. bursa-pastoris*	*rho/het*	Neuffer & Neuffer	4
1394	RCH	Nationalpark Torres del Paine	−52,18	−73	46	*C. bursa-pastoris*	*heteris*	Neuffer & Neuffer	4
1397	RA	Perito Moreno	−50,47	−73	500	*C. bursa-pastoris*	*ten/rho*	Neuffer & Neuffer	4
1412	RA	Las Lenas	−35,18	−69,9	2232	*C. bursa-pastoris*	*rho/het*	Hilger, H.	4
1461	N	Lom	61,83	8,55	400	*C. bursa-pastoris*	Not scored	Neuffer, B.	3
1475	RUS	Almejewsk	54,87	52,3	130	*C. bursa-pastoris*	*rhomboidea*	Neuffer, B.	2
1481	USA	Hobson	47	−110	1306	*C. bursa-pastoris*	*rho/het*	Hellwig, F.	4
1513	USA	Washington	38,9	−77,02	14	*C. bursa-pastoris*	*rhomboidea*	Desmarowitz, C.	4
1514	USA	Shenandoah	38,48	−78,62	315	*C. bursa-pastoris*	*simplex*	Desmarowitz, C.	4
1515	USA	Shenandoah	38,48	−78,62	315	*C. bursa-pastoris*	*simplex*	Desmarowitz, C.	4
1517	USA	New York	40,72	−74,02	0	*C. bursa-pastoris*	*sim/ten*	Desmarowitz, C.	4
1518	USA	New York	40,72	−74,02	0	*C. bursa-pastoris*	*tenuis*	Desmarowitz, C.	4
1519	USA	New York	40,72	−74,02	0	*C. bursa-pastoris*	*tenuis*	Desmarowitz, C.	4
1520	USA	New York	40,72	−74,02	0	*C. bursa-pastoris*	*heteris*	Desmarowitz, C.	4
1530	RUS	Kem	64,97	34,65	10	*C. bursa-pastoris*	Not scored	Hurka, Linde, Neuffer	3
1570	RUS	Uzunovo	54,53	38,62	150	*C. bursa-pastoris*	*rhomboidea*	Hurka, Neuffer, Pollmann	2
1570	RUS	Uzunovo	54,53	38,62	150	*C. bursa-pastoris*	*heteris*	Hurka, Neuffer, Pollmann	2
1581	EC	Quito	−0,22	−78,5	2850	*C. bursa-pastoris*	*simplex*	Hurka, H.	4
1583	EC	Cuenca	−2,83	−79,15	3100	*C. bursa-pastoris*	*simplex*	Hurka, H.	4
1584	EC	Provinz Chimborazo	−1,53	−78,8	3800	*C. bursa-pastoris*	*sim/het*	Hurka, H.	4
1586	EC	Pillaro	−1,17	−78,53	2843	*C. bursa-pastoris*	*simplex*	Hurka, H.	4
1622	NZ	Double Hill	−43,62	171,63	433	*C. bursa-pastoris*	*simplex*	Hurka, H.	4
1643	ZA	Clanwilliam	−32,22	19,2	509	*C. bursa-pastoris*	*sim/het*	Neuffer, B.	4
1648	ZA	Richtersveld	−29,25	17,73	358	*C. bursa-pastoris*	*simplex*	Neuffer, B.	4
1650	ZA	Goageb	−28,02	18,75	932	*C. bursa-pastoris*	*simplex*	Neuffer, B.	4
1652	ZA	Seeheim	−28,75	19,3	932	*C. bursa-pastoris*	*simplex*	Neuffer, B.	4
1655	ZA	Pofadder	−28,75	20,55	995	*C. bursa-pastoris*	*simplex*	Neuffer, B.	4
1668	ZA	Bergwater	−33,58	22,2	1176	*C. bursa-pastoris*	*heteris*	Neuffer, B.	4
1678	ZA	George	−33,95	22,45	223	*C. bursa-pastoris*	*simplex*	Neuffer, B.	4
1682	ZA	Caledon	−34,47	19,9	242	*C. bursa-pastoris*	*simplex*	Neuffer, B.	4
1759	USA	Phoenix	33,4	−111,83	374	*C. bursa-pastoris*	*simplex*	Hurka, H.	4
1763	USA	Coolidge	32,98	−111,53	434	*C. bursa-pastoris*	*simplex*	Hurka, H.	4
1764	USA	Rockwood	33,07	−115,52	−55	*C. bursa-pastoris*	*simplex*	Hurka, H.	4
1996	RCH	Coyhaique	−45,57	−72,07	15	*C. bursa-pastoris*	*heteris*	Klotz, St.	4
2030	CDN	Vancouver	49,27	−122,88	9	*C. bursa-pastoris*	*tenuis*	Hameister, S.	4
2069	MA	Marrakesch	31,63	−7,98	470	*C. bursa-pastoris*	Not scored	Hurka, H.	3
2072	MA	Boulmane	31,32	−6	1800	*C. bursa-pastoris*	Not scored	Hurka, H.	3

### Analysis 2: chlorophyll fluorescence and CO_2_ gas exchange analysis

To test whether progenies from various environmental habitats differ in their ability for their photosynthetic activity, we analysed chlorophyll fluorescence and CO_2_ gas exchange in combination with morphological and anatomical features (Fig. 2, blue colour; Table 1). Of each accession (see Fig. 2) three individuals were grown in a growth chamber with 12-h photoperiod and 15 °C day and 5 °C night temperature. For each individual, the thickness of the leaf and the epidermis cells, stomata density and leaf area were measured.

Using a FluorCam 800MF (Photon Instruments, Brno, Czech Republic), we determined different chlorophyll fluorescence emission parameters of the whole rosette: *F*_m_: maximum fluorescence emission in light; *F*_0_: ground fluorescence in light; *F*: fluorescence emission in light after light pulses of 6000 µmol m^−2^ s^−1^ (for 800 ms every 30 s). From these measured parameters, the photosynthetic light utilization was estimated (Schreiber *et al.* 1986; Genty *et al.* 1989; Scheibe *et al.* 2005; Hanke *et al.* 2009; Scheibe and Dietz 2012; Silva *et al.* 2012; Voss *et al.* 2013).

Furthermore, the CO_2_ gas exchange was measured using the Lic400XT Portable Photosynthesis System (Li-Cor Biosciences, Lincoln, NE, USA). *A*/*C*_i_ curves enabled us to calculate the efficiency of RubisCO to fix CO_2_ under limiting conditions. To determine the photosynthetic capacity of the secondary reaction in relation to the specific light intensity, light saturation curves were recorded. From the obtained parameter values the quantum yield for CO_2_ uptake and the light compensation point (LKP) could be calculated.

### Analysis 3: CO_2_ gas exchange analysis under different light stress conditions

In order to test the ability of progenies from different environmental habitats to light stress, we cultivated sister individuals under different conditions and analysed CO_2_ gas exchange in combination with morphological and anatomical features (Fig. 2, purple colour; Table 1).

**Figure 3.**
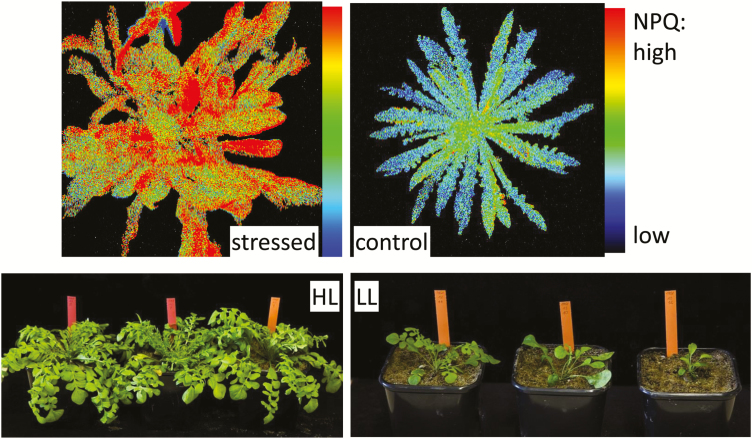
Upper part: non-photochemical quenching (NPQ) of chlorophyll fluorescence as an indicator of energy dissipation in non-stressed (right) and stressed (left) conditions monitored with a FluorCam. Blue: low NPQ, indicating a low proportion of thermal dissipation. Red: high NPQ, indicating a higher proportion of thermal dissipation due to stress. Lower part: individuals of the same age grown under high light (HL, left) and low light (LL, right) conditions, respectively.

We used material from two very different vegetation zones, namely the boreal (1461, 1530) and the meridional (2069, 2072) climatic region to carve out the ecotypic adaptation of the leaves to different environmental conditions (Fig. 2). For each population, up to 49 individuals of the progeny of two individual plants collected in the wild were used. The material was sown in a growth chamber with 12-h photoperiod and 15 °C day and 5 °C night temperature. The material was then divided into four experimental groups: 7.5-h photoperiod, 20 °C, high light setting (800 µmol m^−2^ s^−1^, Fig. 3, left); 7.5-h photoperiod, 20 °C, low light setting (100 µmol m^−2^ s^−1^, Fig. 3, right); 12-h photoperiod, 20 °C, medium light setting (150 µmol m^−2^ s^−1^); 12-h photoperiod, 15 °C day and 5 °C night (‘cold’), medium light setting (100 µmol m^−2^ s^−1^).

The anatomical and physiological analysis was performed as in Analysis 2.

### Analysis 4: thickness of the leaf in a New World transect

As the thickness of the leaf is not only a general character in adaptation to sunny or shady orientation of leaves but seems also to be a character for ecotypic differentiation within Shepherd’s Purse, we performed a large New World transect including populations from South Africa (Fig. 2, orange colour; Table 1). The individuals were grown in a common garden field experiment and planted randomly in the Botanical Garden in Osnabrueck (Germany, May to July 2015). For anatomical analysis, material was taken directly from the field and stored in 70 % alcohol. After 1 day in tap water, the leaves became sufficiently soft for anatomical cuttings. The thickness of five rosette leaves as well as their upper and lower epidermis was determined for the terminal lobe of the leaf and for one lateral leaflet in one to two individuals of each population (Fig. 1). We decided to study different positions of the leaf as the information might differ; also the leaflets may differ between the leaf types (Fig. 1).

### Statistical data evaluation

The data have been analysed statistically with the SPSS software package version 23. To test the normal distribution, we used the Kolmogorov–Smirnov test. In cases where data were significantly not normally distributed, we used the Spearman correlation for the correlation analyses; rho-value and significance are included in the figures. Only significantly correlated data are shown in the figures. The correlations are based on individual data. For testing significant difference (i) between treatments within a population or region, (ii) between populations within one treatment, (iii) between leaf types, we performed parameter-free Wilcoxon-test or the H-test of Kruskal and Wallis (Table 2).

**Table 2.**
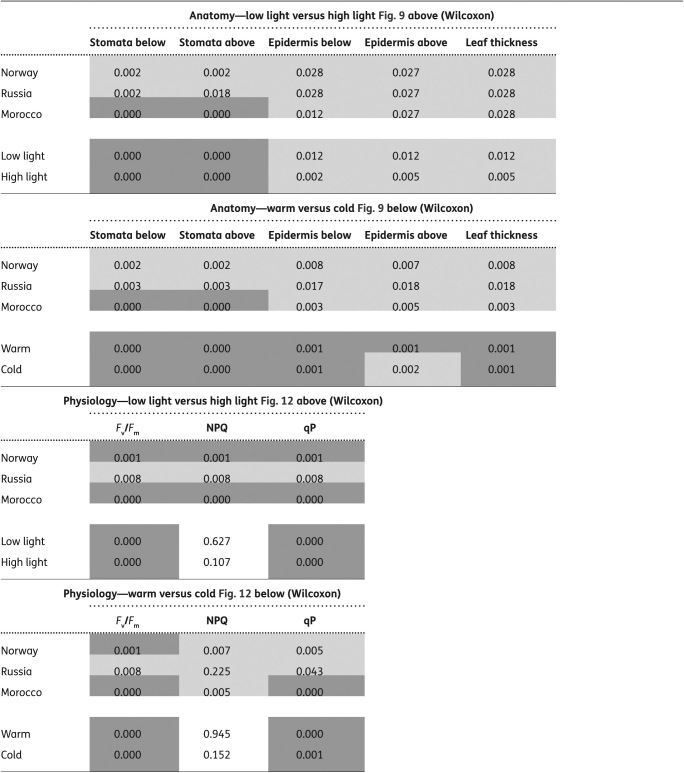

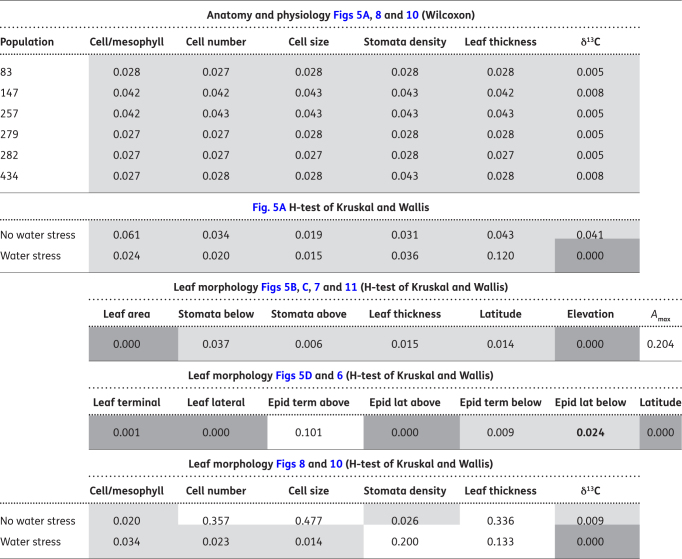
Non-parametric tests for significant differences (Wilcoxon-test, H-test of Kruskal and Wallis). Probability values: *P* < 0.05: significant differences (light grey); *P* < 0.001 highly significant differences (dark grey).

As in Analysis 4, when studying the leaf type of a progeny of 76 populations from various vegetation zones, we performed a *post hoc* Duncan test and an ANOVA.

## Results

In this study, we correlate the results from the four described analyses with the different trait categories anatomy, physiology and morphology. A caveat in these analyses is, however, that we cannot ascertain the similarities of developmental maturation between the leaves analysed. Some of the differences observed, may, therefore, reflect intraspecific variation in life history.

### Anatomical analyses

**Figure 4.**
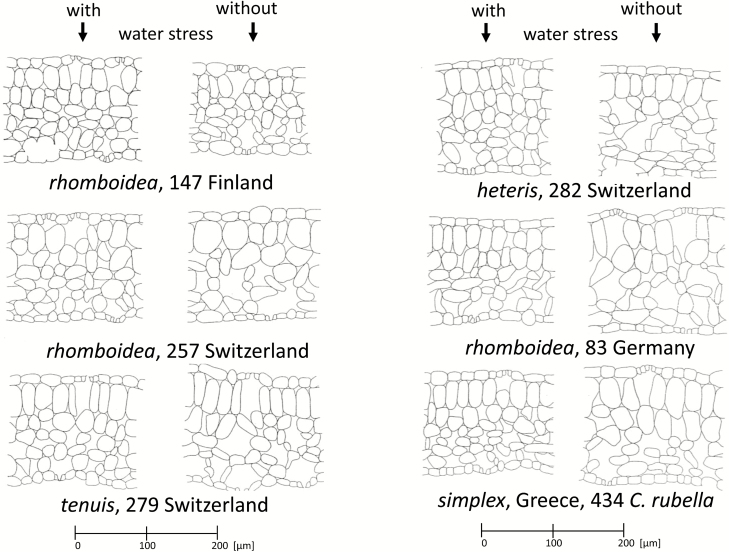
Cross sections of leaves from plants grown under different water stress conditions (Analysis 1). Correlation analyses are given in Figs 5A, 8 and 10.

In Analysis 1, progeny from wild populations, when grown under water stress, developed denser mesophyll cells compared with the loose texture and large intercellular spaces in unstressed plants (Fig. 4). Furthermore, the palisade cells appeared narrower with a smaller diameter. *Capsella rubella* developed two palisade layers under both water-stressed and control conditions (Pop. 434), whereas *C. bursa-pastoris* exhibits two layers only under water stress (e.g. Pop. 147, 282). Pop. 257 showed only one palisade layer in both conditions.

**Figure 5.**
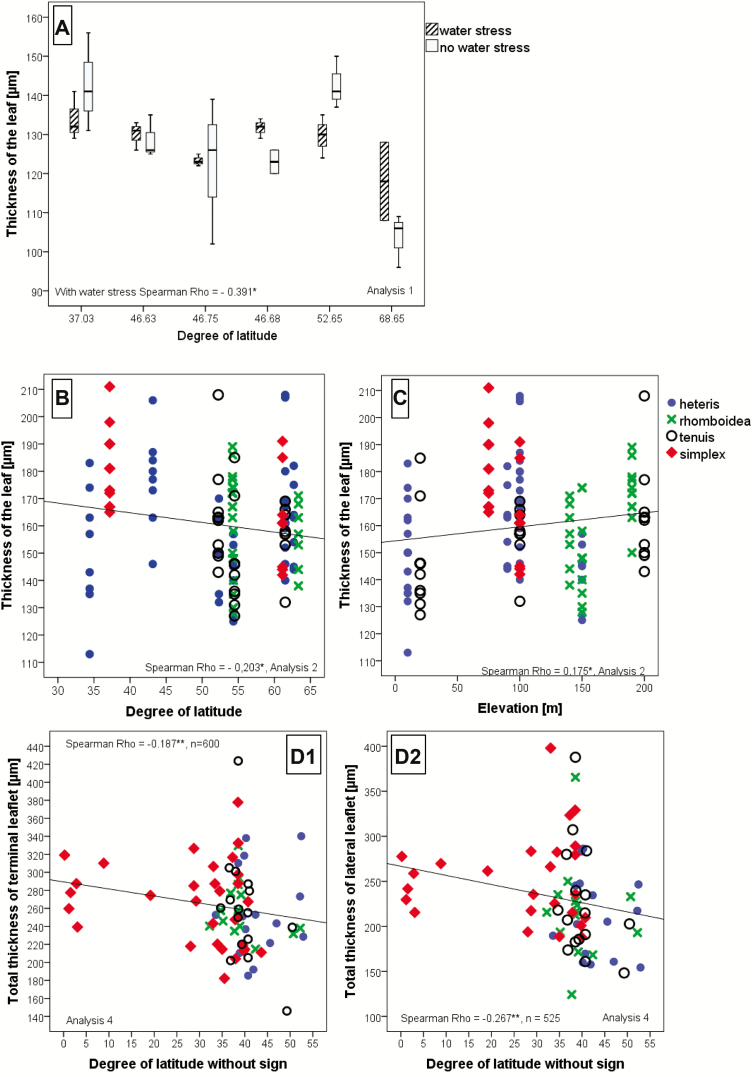
Spearman correlation of leaf thickness with latitude/elevation. The thickness of the leaf is significantly negatively correlated with latitude and in one case positively with the elevation; (A) = 25:15 °C, 9-h photoperiod, populations arranged according to the degree of latitude, three individuals have been tested for each population and treatment, both treatment groups differed significantly or highly significantly when tested by H-test of Kruskal and Wallis (see Table 2); (B and C) = 15:5 °C; 12-h photoperiod; (D) = common garden field experiment (*n* = number of individuals, in the figure are shown mean square values of the populations). Rho-value and significance (*α < 0.05, **α < 0.01) are included in the figures. A pairwise correlation test between the terminal and the lateral leaflet thickness (Kendall-, Friedman- and Wilcoxon-test) evidenced for significant differences between both. Only significantly correlated data are shown in the figures. The correlations are based on individual data.

**Figure 6.**
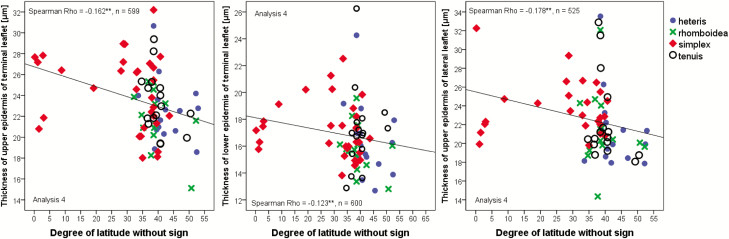
Spearman correlation of epidermis thickness with the degree of latitude. At the terminal leaflet, the upper and the lower epidermis is significantly negatively correlated, at the lateral leaflet only the upper epidermis is significantly correlated with the latitude. As the material originated from North and South America, we evaluated the statistics for the degree of latitude without a signature (north or south of the equator) for calculating a linear correlation. Rho-value and significance (*α < 0.05, **α < 0.01) are included in the figures. Only significantly correlated data are shown in the figures. The correlations are based on individual data, *n* = number of individuals; mean square values of the populations are shown in the figure.

Anatomical leaf parameters of various provenances grown under different conditions were correlated with geographical/elevational parameters at the places of origin (Fig. 5): the whole leaves and, in particular, the epidermis cell layer became significantly thinner with a higher degree of latitude (Fig. 5, all analyses, Fig. 6). In Analysis 4, we differentiated between the terminal leaflet (Fig. 5D1) and the lateral leaflet (Fig. 5D2) and observed at both positions that the thickness was the same. Interestingly, with a higher elevation at the place of origin leaves became thicker in Analysis 2 (Fig. 5C).

**Figure 7.**
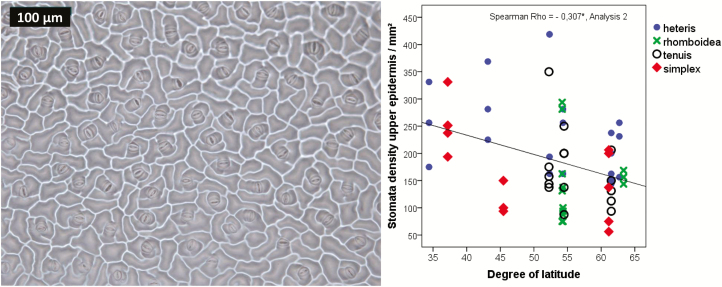
Spearman correlation of stomata density with latitude in Analysis 2 (15:5 °C, 12-h photoperiod). An example of a nail varnish imprint of the lower epidermis (left) used for the measurements is shown. Rho-value and significance (*α < 0.05, **α < 0.01) are included in the figures. Only significantly correlated data are shown in the figures. The correlations are based on individual data.

**Figure 8.**
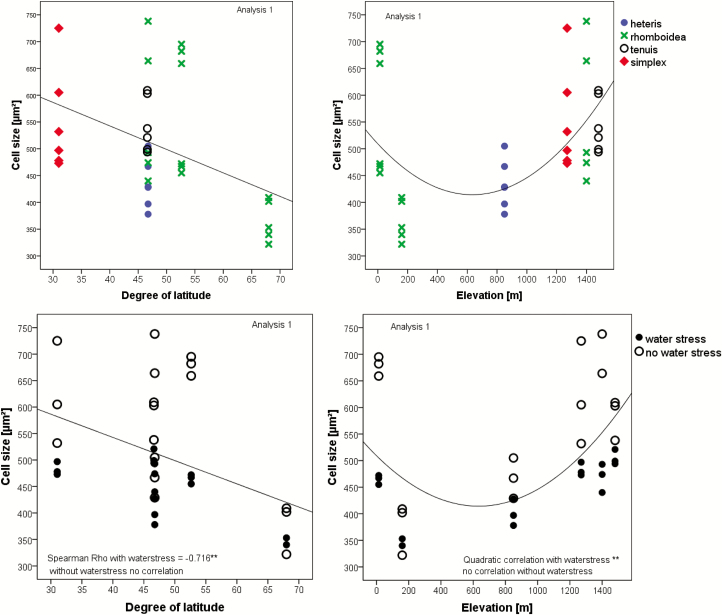
Correlation of cell size with latitude (linear, Spearman) and altitude (quadratic). The correlation is highly significant under water stress conditions. Upper diagram: labels according to the leaf type; lower diagram: same correlation, but labels according to watering conditions (Analysis 1). Rho-value and significance (*α < 0.05, **α < 0.01) are included in the figures. Both treatment groups differed significantly when tested by Wilcoxon-test (see Table 2). Only significantly correlated data are shown in the figures. The correlations are based on individual data.

Even the epidermis layer itself varied with the degree of latitude and became thicker for populations originating from locations closer to the equator (Fig. 6). Stomata became less dense when populations originated from northern latitudes (Fig. 7). Cell sizes appear to decrease with the degree of latitude (Fig. 8, left) and increase with higher elevation (Fig. 8, right). However, only when grown under water stress conditions was the correlation highly significant.

**Figure 9.**
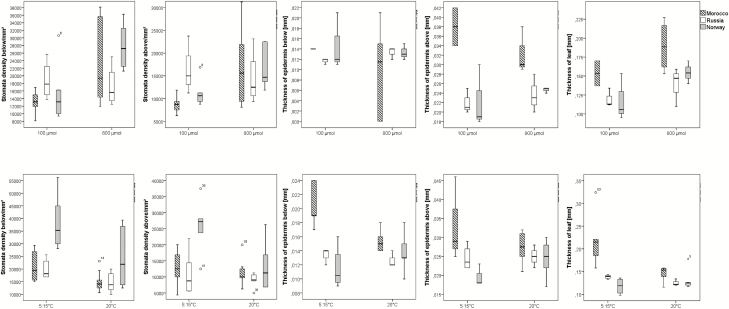
Anatomical data of provenances from different climatic/geographic regions under low (100 μmol m^−2^ s^−1^ ) versus high (800 μmol m^−2^ s^−1^ ) light conditions (upper part: 7.5-h photoperiod, 20 °C, Analysis 3), and cold versus warm temperatures (lower part: 12-h photoperiod, 150 µmol quanta/m^2^ s, Analysis 3), three individuals for each provenance and treatment. All treatment groups differed significantly or highly significant when tested by Wilcoxon-test (see Table 2).

Comparing populations originating from very divergent local conditions: with dry and hot conditions in the summer and high irradiation in Morocco, and temperate humid conditions and very long days in summer in Norway and in Karelia (Russia) (Fig. 9), the population from Morocco possessed thicker leaves and larger upper epidermis cells under all conditions, compared to the other populations. Whereas the Russian population showed a low variation of stomata density when grown under low or high light conditions and between cold versus warm temperature, respectively, the populations from Norway and Morocco increased stomata density when grown under high light and in cold conditions.

### Physiological analyses

**Figure 10.**
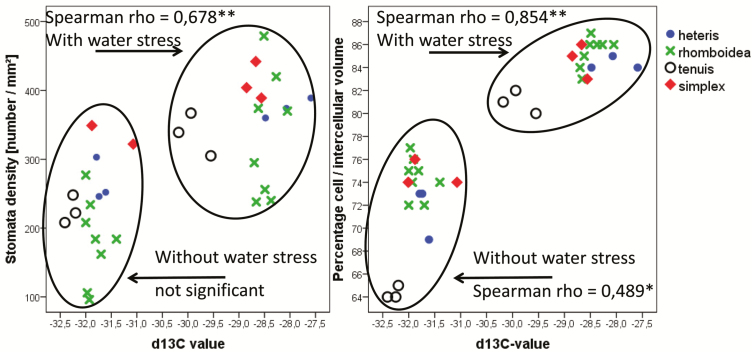
Spearman correlation of δ^13^C values and anatomical traits. The δ^13^C values increased under water stress and were highly significantly correlated with anatomical traits under water stress conditions. The leaf type *tenuis* is characterized by lower δ^13^C values under both conditions compared with the other types (see 25:15 °C, 9-h photoperiod). Except for *simplex* leaf type (*Capsella rubella*), all individuals belong to *Capsella bursa-pastoris*. Rho-value and significance (*α < 0.05, **α < 0.01) are included in the figures. Only significantly correlated data are shown in the figures. Without *C. rubella* (phenotype *simplex*) only ‘percentage cell/intercellular volume: δ^13^C’ under water stress remained significant (Spearman rho = 0.635*). Both treatment groups differed significantly or highly significant when tested by Wilcoxon-test (see Table 2). The correlations are based on individual data.

With water stress, the δ^13^C values were higher, meaning that more carbon isotopes had been fixed and assimilated during photosynthesis. As RubisCO discriminates the isotopes in the case of unhampered CO_2_ uptake from the atmosphere into the intercellular spaces and across the cell membranes, higher values are a result of partially closed stomata and increase CO_2_ isotope concentration within the leaf. With water stress, the proportion of cell volume to intercellular volume increased (consequently the intercellular space decreased, Fig. 10), and the stomata density also increased (see Fig. 7). Although the differences between water-stressed and non-stressed individuals are apparent in leaf anatomy (Fig. 4) and physiology (Fig. 10), leaf anatomy and δ^13^C values clearly differ between the provenances. The *tenuis* leaf type (provenance from the Alps) differed clearly from the other provenances by larger intercellular space compared to the cell volume. This trait coincided with lower δ^13^C values which might be the result of reduced ability to close stomata under water stress conditions. The difference between the δ^13^C values under water stress conditions might hint at an ecotypic differentiation with a high phenotypic plasticity for the provenances with the other leaf types (*heteris*, *rhomboidea* and *simplex*). The *C. rubella* individuals showed the highest stomata density without water stress. With water stress, these individuals intermingled in between the *C. bursa-pastoris* individuals, so that no differentiation between the two species was apparent from the characterized parameters. When excluding *C. rubella* from the analysis, only the correlation between the percentage cell/intercellular volume versus δ^13^C under water stress condition remained significant (Spearman rho = 0.635*). To substantiate differences between the two species, which are often to be found in mixed populations in their common distribution area, analysis of a larger number of populations is needed.

**Figure 11.**
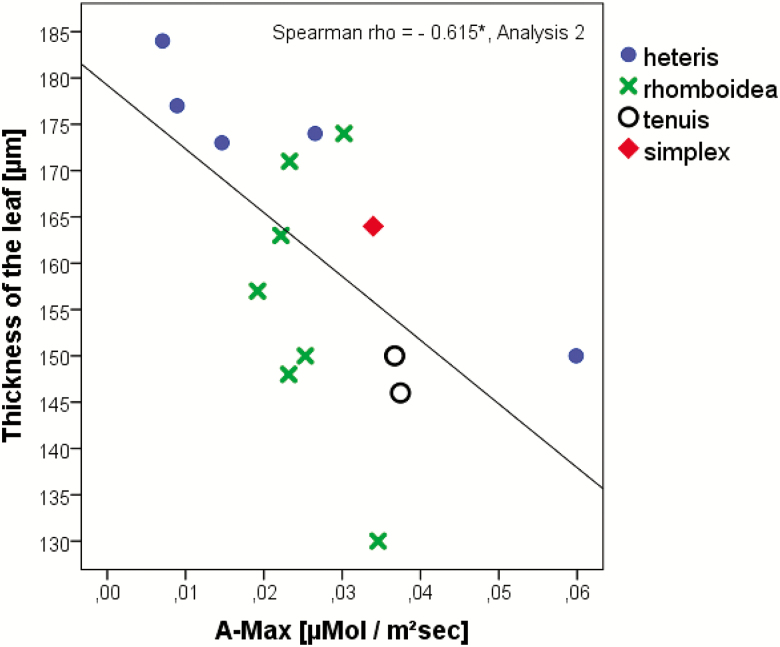
Spearman correlation of leaf thickness with CO_2_-fixation index. This value represents the CO_2_-fixation index, i.e. the maximal rate of CO_2_ assimilation (*A*_max_) at saturating light (Analysis 2). Rho-value and significance (*α < 0.05, **α < 0.01) are included in the figures. Only significantly correlated data are shown in the figures. The correlations are based on individual data.

**Figure 12.**
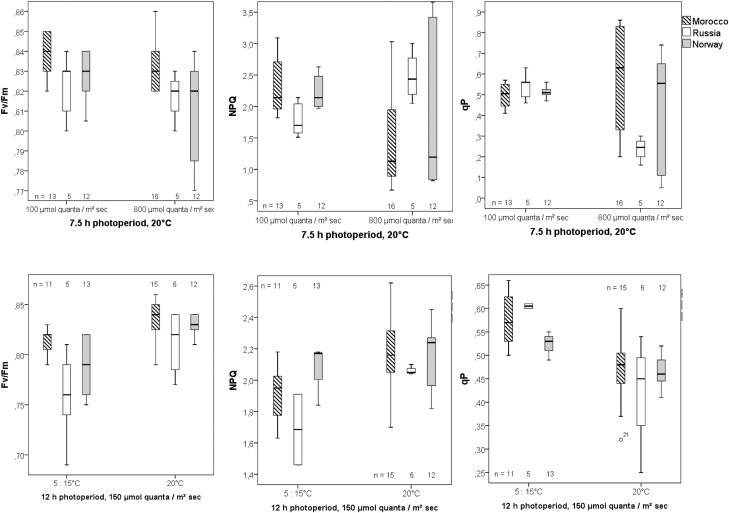
Chlorophyll fluorescence parameters of provenances from three different regions under low (100 µmol m^−2^ s^−1^) versus high (800 µmol m^−2^ s^−1^) light conditions (upper diagrams: 7.5-h photoperiod, 20 °C, Analysis 3), and low versus higher temperatures (lower diagrams: 12-h photoperiod, 150 µmol m^−2^ s^−1^, Analysis 3). Parameters were measured under steady-state light conditions. *F*_v_/*F*_m_ = maximum quantum yield of PSII; NPQ = non-photochemical quenching; qP = photochemical quenching (*n* = number of individuals tested). Most treatment groups differed significantly or highly significant when tested by Wilcoxon-test (see Table 2).

The CO_2_-assimilation rate correlated highly significantly with the thickness of the leaf. Populations originating from higher latitudes develop thinner leaves under greenhouse conditions (Fig. 5B), enabling higher CO_2_-assimilation rates (Fig. 11). The non-photochemical quenching (NPQ) of the Russian population increased significantly under high light which might indicate higher stress from the increased temperature for these plants (Fig. 12). On the other hand, the NPQ of the Moroccan individuals is even lower under high light conditions, suggesting that these conditions are tolerated easily by these individuals (Fig. 12). The NPQ values are highly significantly negatively correlated with the stomata density at the lower surface (Spearman rho = 0.352**), namely, the stomata density increased at higher NPQ values. Efficient light use for CO_2_ assimilation as can be recognized by photochemical quenching (qP) was highest in the Moroccan population under high light conditions, whereas the Russian population was characterized by low qP values (Fig. 12). Under all other environmental conditions, the populations displayed barely any differences. The qP values are significantly positively correlated with the stomata density at the lower leaf surface (Spearman rho = 0.284*) and with the area of a rosette leaf (Spearman rho = 0.281*).

### Leaf types

The geographical distribution of the Mendelian leaf types according to Shull is apparent when regarding the measured leaf thickness from accessions along a transect through North and South America (Fig. 6): the *simplex* leaf type occurred more frequently close to the equator and seemed to be nearly absent at higher latitudes. This appears to be confirmed in the isotope analysis (Fig. 8), whereas, in order to be able to make a clear statement, the number of studied populations in Analysis 1 is too small. On the other hand, in Analysis 2, this leaf type *simplex* did not correlate with latitude. In all analyses, the *tenuis* leaf type prefers temperate regions with adequate humidity during the vegetation period, even at higher altitudes (Analysis 1, Fig. 8). The reduced plasticity of *tenuis* compared with the other leaf types is also evident for the physiological traits, as the δ_13_C values were considerably lower compared with the values of the other leaf types, especially under water stress conditions (Analysis 1, Fig. 10). However, in Analysis 1, the number of analysed individuals and populations was restricted, and therefore, a higher sample number is necessary to verify our interpretation.

To summarize and generalize the results of our observations and experimental studies, the following statements are put forward:

- The thickness of the leaf, of the epidermis and the epidermal cell size are negatively correlated with the degree of latitude.
- The stomata density varies significantly between different light conditions and provenances.
- Physiological studies (δ^13^C values) showed that the leaf types/ecotypes *heteris*, *rhomboidea* and *simplex* appear to be able to close the stomata more efficiently under water stress conditions than the *tenuis* leaf type which might be due to lower plasticity.
- Ecotypes with thinner leaves exhibit a lower maximal rate of CO_2_ assimilation (*A*_max_) at saturating light.
- Physiological parameters resulting in high photosynthetic capacity under stressful, strong light conditions are typically found when the plants originate from hot, dry and sunny regions.

## Discussion

The high degree of polymorphism of the leaves in the genus *Capsella* has been well known for more than 100 years. Almquist (1907, 1921) listed 200 elementary species, and in his opinion, this is a result of high variability of the genus in nature. In parallel, the geneticist Shull (1909, 1911) performed extensive inheritance studies which formed the basis for the hypothesis of the existence of two Mendelian loci with two alleles, each responsible for the four basic leaf types within the genus. Later on, Shull argued in favour of an additional factor ‘I’ for leaves with completely entire margins (‘*simplissima*’, Shull 1929). Particular plants with small rosettes of linear leaves which have a spider-like appearance have been designated by Hus (1914) as x*Capsella bursa-pastoris arachnoidea*, and a leathery appearance corresponds to the dominant allele ‘*K*’ which was named ‘*coriacea*’ factor by Shull (1929). Clausen and Hiesey (1958) confirmed at least four pairs of genes responsible for the leaf shape in *Capsella* and suggested even a higher number of loci that are responsible for other modifications of the leaf. Our observations evidenced that, in the case of heterozygotes of tetraploid *C. bursa-pastoris* individuals, the dominance might be incomplete (e.g. *AaaaBbbb*) and then the leaf type would be intermediate and not distinguishable.

**Figure 13.**
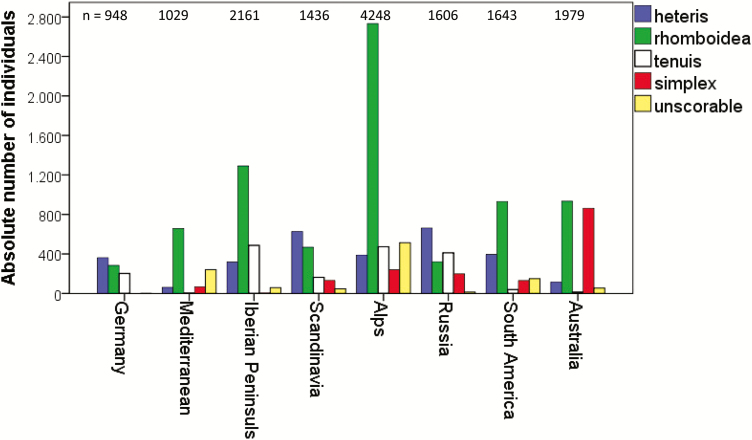
Distribution of rosette leaf types in various regions worldwide, *n* = number of individuals.

So far, we determined rosette leaves of many provenances worldwide (Fig. 13). In one case regarding provenances growing along an altitudinal cline, we detected an increase in the percentage of the *B*-allele with higher elevation (Neuffer and Bartelheim 1989). The *B*-allele is responsible for dividing the lobes to the midrib in the leaf types *heteris* and *rhomboidea*. In another analysis with provenances growing along a latitudinal cline, the variability in leaf types did not correspond to the north–south gradient. In general, out of 15 050 scored leaves (Fig. 13), 19 % have been *heteris*, 51 % *rhomboidea*, 12 % *tenuis*, 11 % *simplex* and 7 % remained unscored.

The question is: why are the leaf types not randomly distributed in the case of selection neutrality and why is the percentage of the *rhomboidea* leaf type high? Are the leaf types adaptive by themselves, is the adaptation mirrored by anatomical or physiological characters, or is the leaf type linked to adaptive anatomical and/or physiological leaf characters? Dissection of the leaf has been described as being inversely correlated with the mean annual temperature at the community and species level for trees (Royer *et al.* 2009). Therefore, dissection has been used for paleoclimatic reconstructions (Royer *et al.* 2005; Little *et al.* 2010), and it is conceivable that the dominance of the *rhomboidea* leaf type is a consequence of better adaptation of dissected leaves to various climates. In *Capsella*, the morphology of the leaf type depends for some reason on the environmental conditions. *Capsella* appears to exhibit earlier flowering times the longer the day in a long-day photoperiod (Hurka *et al.* 1976; Neuffer 1990). Under long day and warm temperature conditions some ecotypes flower so early that only a few rosette leaves are able to develop (Neuffer and Hurka 1986), resulting in these leaves which do not attain a pronounced leaf morphology but remain simple (Neuffer 1989). When grown under short day conditions and/or cold temperatures these provenances produce more rosette leaves (Neuffer and Hurka 1986), which enables them to reach the state which facilitates the development of the more pronounced leaf types (Neuffer 1989). In our study, the distribution of the leaf types was clearly divided into two subgroups according to the climax vegetation zone or the thermal vegetation zone according to Schroeder (1998) documented by the Duncan test in Analysis 4 (Table 3): regarding climax vegetation, only the leaf type *tenuis* belonged to a second subgroup, whereas regarding thermal vegetation zones, both *tenuis* and *simplex* comprised a second subgroup.

**Table 3.** ANOVA and *post hoc* Duncan test to prove leaf type distribution to climax vegetation zones and thermal vegetation zones (Schroeder 1998, Analysis 4). Probability for subgroups *α* = 0.05.

Leaf type	*N*	Climax vegetation zone		Thermal vegetation zone	
		Group 1	Group 2	Group 1	Group 2
*heteris*	125	4.72		2.92	
*rhomboidea*	90	4.67		3.00	
*tenuis*	125		5.92		3.40
*simplex*	260	4.25			3.35
Significance		0.224	1.000	0.518	0.663

The molecular basis of the leaf shape in Brassicaceae is beginning to be unravelled. At first, the leaf shape seems to have evolved from small, simple leaves (*Aethionema* spec.) to compound leaves (*Cardamine* spec.). First results for *Cardamine hirsuta* have been obtained in the Tsiantis group: they hypothesize that 44 genes are potentially implicated in the leaf development, e.g. *SHOOT MERISTEMLESS*, *BREVIPEDICELLUS* or *CUP*-*SHAPED COTYLEDON* (Gan *et al.* 2016). For the leaflet formation in comparison with the simple leaves of *Arabidopsis thaliana*, the enrichment of the transcription factors of the *PLETHORAS* family is required, especially of *PLT*7 (Gan *et al.* 2016). Furthermore, Hay and Tsiantis (2016) detected a duplication of the *gene LATE MERISTEM IDENTITY*1 (*LMI*1) giving rise to *REDUCED COMPLEXITY* (*RCO*) in *C. hirsuta*. This duplication is lost again in *A. thaliana* and seems to be responsible for the reversal to simple leaves. In a detailed analysis of Sicard *et al.* (2014) with the two diploid species *Capsella grandiflora* and *C. rubella*, a second duplication which forms *RCO*-*A* and *RCO*-*B* has been detected. The difference between *C. grandiflora* with simple leaves (leaf type *simplex*) and *C. rubella* with dissected leaves (leaf type *rhomboidea*) was an allelic variation at the *RCO*-*A* locus. Furthermore, these authors detected four insertions of relatively recent origin in the *RCO*-*A* genomic organization which differed either in their absence or presence in various provenances. One future aim is to identify the molecular genetic background for the above-mentioned, already known genes and alleles that model the morphology of *Capsella* rosette leaves.

Finally, the adaptation of the rosette leaf is of highest importance, especially in the case of late flowering to biennial ecotypes overwintering with a rosette. In general, *C. bursa-pastoris* forms larger rosette leaves in later flowering plants under field conditions in common garden experiments (e.g. Neuffer and Hurka 1986; Neuffer 2011).

In this study, it is the first time that we report anatomical and physiological results of Shepherd’s Purse leaf types. Körner (2003) reviewed leaf anatomical and physiological characters and discussed how leaves are adapted to high mountain ecosystems. He observed a significantly thicker mesophyll and larger epidermis cells for plants from higher altitudes which is in accordance with our findings. Regarding the fact that the climate in high latitudes of Scandinavia might be similar to high elevations in the Alps, the result for *Capsella* seems to be contradictory at first glance. However, in more northern latitudes, the days in the summer are longer and irradiation less strong. Therefore, the occurrence of thinner leaves with smaller epidermis cells in northern latitudes can be explained as a logical adaptation.

The physiological adaptation of leaves to various environments is often characterized by WUE, as can be deduced from δ^13^C values. In a comparison between different provenances of the grass *Leymus chinensis* from dry steppe regions of Asia, the differentiation under various conditions seemed to be more the result of plasticity rather than of ecotypic differentiation (Liu *et al.* 2016). The authors argue with the clonal propagation of this species which comes close to the general-purpose genotype in the sense of Baker (1974). In our case, the differentiation is apparently not only plastic but also ecotypic with a genetic background. We assume that ecotypes when growing under high light intensities at their places of origin are more adapted to high light, and are able to increase their quantum-yield efficiency considerably, whereas non-adapted genotypes are not able to do so or even suffer from photoinhibition as can be deduced from maximum quantum yield of PSII (*F*_v_/*F*_m_) values when analysed with the FluorCam. This ecotypic differentiation might be the result of the mixed mating system with an outcrossing of up to 12 % under good field conditions (Hurka *et al.* 1989), whereas *L. chinensis* is a clonally propagating species (Liu *et al.* 2016).

Another aspect of the adaptation of the leaf are the qualitative and quantitative intraspecific variations of the main flavonoid pattern which was put forward by Eschmann-Grupe (1990) with populations of *C. bursa-pastoris*. The leaves appeared to reflect the adaptation of a population to the place of origin and varied with different environmental conditions. Five main and nine less prominent flavonoids were detected. Focusing on the main flavonoids the authors studied one population from high altitudes in the Alps, one from Norway and one from central Germany under various conditions in the growth chamber as well as in a reciprocal field experiment in 2000 m elevation in the Alps and in central Germany. The three populations varied qualitatively in their flavonoid composition only the population from the Alps contained all five main flavonoids. The Norwegian population contained no isoorientin, and the population from central Germany lacked diosmetin-7-O-β-D-glucoside. Under the various environmental conditions, the pattern did not differ qualitatively, but the quantity was increased significantly in field conditions, especially in the Alps. The composition and amount of secondary metabolites stored in the vacuoles of epidermal cells might be another physiological adaptation of *Capsella* ecotypes to high irradiation. These characteristics are possibly interesting for further studies of the ecotypic differentiation of *Capsella* rosette leaves besides the morphological, anatomical and photosynthetic parameters used in this study.

## Conclusion

Here, we present a first insight into the ecotypic differentiation of *Capsella* rosette leaves in a combination of morphological, anatomical and physiological characters. The geographical distribution and frequencies of specific morphological leaf types seem to mirror the adaptation to particular environmental conditions at the places of origin. However, the actual adaptation might be overlaid by anatomical and physiological adaptive traits which, with the numerous combinations of variations, point to a genetic background. To unveil the molecular background of the ecotypic differentiation of the *Capsella* rosette leaves, the knowledge of the molecular settings behind the leaf morphology is not yet sufficient. The clear geographic distribution pattern of the morphological leaf types might be partially adaptive by itself and therefore responsible for the frequency differences in various regions. However, the selection for specific morphological leaf types under different environmental conditions could be caused by genetic hitchhiking via anatomical or physiological adaptive characters with a genetic background. This linkage between the various traits is possible via closely linked loci on the same chromosome, and the effect is enhanced tremendously by the mating system which relies predominantly on selfing. It is, therefore, necessary to phenotypically and genetically elucidate all three aspects in combination: morphology, anatomy and physiology.

## Sources of Funding

Financial support for expeditions to collect plant material was given in the framework of various projects funded by the DAAD, DFG and BMBF. Support for Analysis 4 was provided by DFG (NE 314/11-2).

## Contributions by the Authors

B.N. provided the material, supervised Analyses 1 and 4 as well as the anatomical and morphological part of Analyses 2 and 3, evaluated data and wrote draft versions of the manuscript. C.W. supervised Analysis 4 which is part of her PhD thesis, evaluated data and contributed to draft versions of the manuscript. I.V. performed the physiological lab work of Analyses 2 and 3, and evaluated and interpreted these data. R.S. supervised the physiological part of Analyses 2 and 3, and wrote parts of the manuscript.

## Conflict of Interest

None declared.
